# High-frequency repetitive transcranial magnetic stimulation alleviates the cognitive side effects of electroconvulsive therapy in major depression

**DOI:** 10.3389/fpsyt.2022.1002809

**Published:** 2022-10-03

**Authors:** Xing Chen, Tongtong Zhang, Xiaoyan Shan, Qun Yang, Peiyun Zhang, Haijiao Zhu, Fei Jiang, Chao Liu, Yanzhong Li, Weijun Li, Jian Xu, Hongmei Shen

**Affiliations:** ^1^Laboratory of Biological Psychiatry, Nantong Mental Health Center & Nantong Brain Hospital, Nantong, China; ^2^Key Laboratory of Neuroregeneration of Jiangsu and Ministry of Education, Co-innovation Center of Neuroregeneration, Nantong University, Nantong, China

**Keywords:** repetitive transcranial magnetic stimulation (rTMS), electroconvulsive therapy, depression, anxiety, cognitive impairment

## Abstract

**Objective:**

The retrospective study aimed to explore the difference in mood outcomes and cognitive function between high-frequency repetitive transcranial magnetic stimulation (HF-rTMS) over dorsolateral prefrontal cortex (DLPFC) and electroconvulsive therapy in major depression disorder (MDD) patients and to examine the improvement of HF-rTMS on cognitive impairment evoked by electroconvulsive therapy (ECT).

**Materials and methods:**

A total of 116 participants with MDD, who completed a 4-week follow-up assessment, were enrolled. The cohort consisted of 26 cases classed as control, 46 participants administrated with HF-rTMS (HF-rTMS group), 22 patients treated with ECT (ECT group), and 23 cases treated with HF-rTMS and ECT at the course of hospitalization (HF-rTMS + ECT group). Medication was kept constant as well in all participants. The 17-item Hamilton Depression Rating Scale for Depression (HAMD-17) and 14-item Hamilton Anxiety Rating Scale (HAMA-14) were used to assess depression and anxiety, respectively. Montreal Cognitive Assessment (MoCA) was to elevate cognitive function.

**Results:**

No statistical significance was found for baseline in sociodemographic, characteristics of depression, anxiety and cognition, and psychopharmaceutic dosages among control, HF-rTMS, ECT, and HF-rTMS + ECT groups (*p* > 0.05). Compared with baseline level, total scores of HAMD-17 and HAMA-14 significantly decreased at the end of 4 weeks after treatment (*p* < 0.001). Furthermore, the decline in scores of HAMD-17 and its sleep disorder and retardation factors from baseline to post-treatment was greater in HF-rTMS, ECT, and HF-rTMS + ECT group than in control (*p* < 0.05), and there was a significant difference between control and HF-rTMS group in the decline of psychological factor of HAMA-14 (*p* < 0.01). ECT treatment evoked total score of MoCA to decrease significantly at the end of 4-week after intervention (*p* < 0.001), and the decline in scores of MoCA and its delayed recall and language performances from baseline to post-treatment was greater in ECT than control, HF-rTMS, and HF-rTMS + ECT (*p* < 0.05).

**Conclusion:**

High-frequency repetitive transcranial magnetic stimulation improved psychological anxiety and ameliorated the cognition impairment evoked by ECT though it had the same anti-depressant efficacy as ECT.

## Introduction

Major depressive disorder (MDD) is one of the most prevalent mental illness, affecting an estimated 300 million people worldwide (1–3). MDD impairs social functioning, causes personal suffering, and has economic consequences for the individual and society due to long-term incapacitation and loss of production (4). Coronavirus disease 2019 (COVID-19)-related lockdown impacted on lifestyle habits and behavioral suicide risk factors and worsened mental health of the populations, especially depression, in different regions (5–8). Monoamine hypothesis offers the most reliable explanation for the development of MDD (9), selective serotonin reuptake inhibitors (SSRIs) and serotonin and noradrenaline reuptake inhibitors (SNRIs) are the first-line drugs for the treatment of MDD (10), and duloxetine, as a potent SNRI, has similarly effective as various SSRIs (11). In addition, dietary capsaicin confers the prevention of depression *via* regulation of the monoamine transmitter by improving the gut microbiota dysbiosis (12–14). However, non-invasive brain stimulation (NIBS) is one of the fastest-growing fields in the treatment for major depression (15), and it refers to a set of techniques used to modulate brain activity using non-implantable methods, including electroconvulsive therapy and transcranial magnetic stimulation (TMS) (16, 17).

Electroconvulsive therapy (ECT) is the first and the most effective clinical trial as a NIBS technique in the field of neuropsychiatry, and electrodes are mostly placed bilaterally over the temporal cortex. It has been demonstrated that ECT has remission rates >70% for treatment of depression, involving an electrical current being passed through the brain to induce a generalized seizure (18–20). However, ECT is limited due to the need for infrastructure, social stigma, and potential cognitive side effects, including postictal disorientation, anterograde amnesia, retrograde amnesia, and impairments in multiple other cognitive domains, including verbal fluency and executive function (21, 22). In particular, the severity and characterization of cognitive impairment is the great debate (17, 23), though the cognitive impairment was minimized by many different treatment modifications over decades (24). In addition to the cognitive impairment, anesthesia and muscle relaxants are needed in ECT procedures though ECT technique has greatly been improved and can safely provide relief for subjects with severe major depression (25, 26).

Transcranial magnetic stimulation is one novel non-invasive approach to MDD over the last decades. The technique utilizes electromagnetic fields to alter neural activity in relatively focal, superficial areas of the brain, and repetitive TMS (rTMS) delivers a series of electromagnetic pulses to modulate the activity of nerve cells in the special regions (27). In MDD patients, functional magnetic resonance imaging (fMRI) data showed the left-right dorsolateral prefrontal cortex (DLPFC) imbalance, which is hypoactivation in the left DLPFC, and hyperactivity in the right DLPFC (28). Based on the finding, rTMS for depression targets the DLPFC (29), and rTMS has been applied using various protocols, including low-frequency (1 Hz) or high-frequency stimulation (10–20 Hz), unilateral or bilateral stimulation (30). Typically, high-frequency stimulation of the left DLPFC is the most common treatment protocol for MDD and has been shown to have a statistically and clinically significant anti-depressant effect (31). However, whether high-frequency rTMS over the left DLPFC has the same anti-depressant effect as ECT is elusive in MDD patients. Therefore, one aim of the retrospective study was to provide evidence for the difference in mood outcomes between HF-rTMS and ECT. On the contrary, patients with MDD can benefit from HF-rTMS in terms of cognition, including memory, and attention (32). Thus, another aim of the current study was to compare the effects of HF-rTMS and ECT on cognitive function and to test whether HF-rTMS could prevent the cognitive impairment caused by ECT. The underlying hypothesis is that HF-rTMS has the same anti-depressive effect as ECT, but it could not evoke cognitive impairment. Furthermore, HF-rTMS could prevent the cognitive impairment caused by ECT in MDD patients.

## Materials and methods

The study was performed in accordance with the Declaration of Helsinki and approved by the Ethics Committee of Nantong Fourth People’s Hospital in China (Approval Number: 2020K007), and this was a retrospective study.

### Participants

**FIGURE 1:**
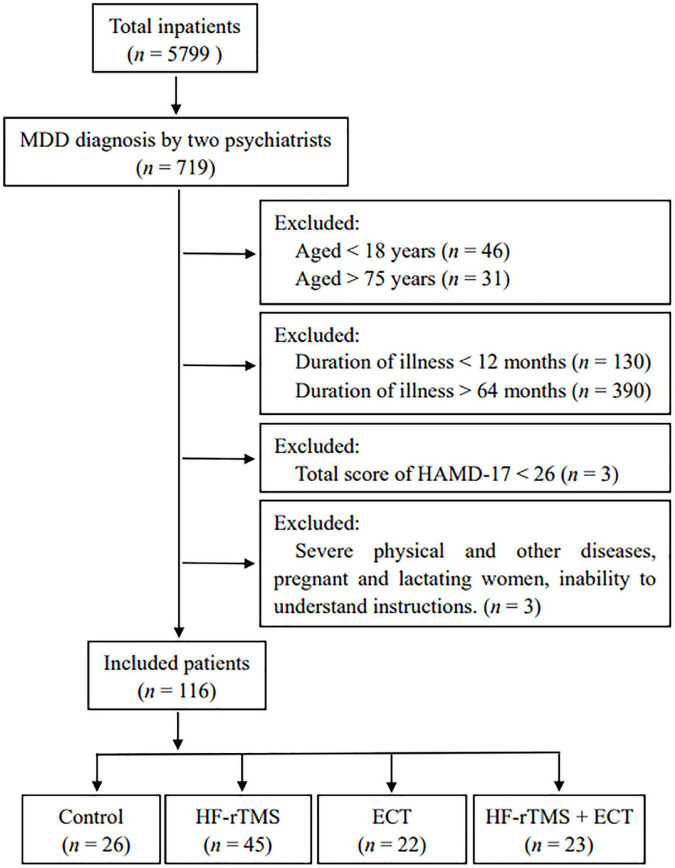
Schematic of the participant flow. MDD, major depressive disorder; HF-rTMS, high-frequency repetitive transcranial magnetic stimulation; ECT, electroconvulsive therapy; HAMD-17, 17-item Hamilton depression rating scale.

One hundred and sixteen inpatients at the Department of Clinical Psychology of Nantong Mental Health Center from November 2017 to December 2019, independently diagnosed with MDD based on the criteria of Diagnostic and Statistical Manual of Mental Disorders, 5th edition (DSM-V) by two psychiatrists, were successfully recruited in the retrospective study. A schematic of the participant flow was shown in Figure 1. Medication was kept constant as well in all participants.

### Repetitive transcranial magnetic stimulation

Repetitive transcranial magnetic stimulation (rTMS) was administered 30 min daily, using the transcranial magnetic stimulator (CCY-I, Yiruide Co., Ltd., Wuhan, China) with circular coil at a diameter of 126 mm. Prior to the first rTMS treatment, the TMS intensity for each patient was determined based on the resting motor threshold (rMT), which evokes the motor potential in the abductor polis brevis muscle. According to rTMS studies for depression, stimulation was applied in left DLPFC, 5 cm anterior to the scalp position for the motor potential. About 1,150 pulses of 10 Hz excitatory TMS were applied over the left DLPFC by 5-s trains with a 35-s inter-train interval.

### Electroconvulsive therapy

Electroconvulsive therapy stimulation was delivered using the Thymatron System IV instrument (Somatics LLC, Lake Bluff, IL, USA) by bidirectional pulse square wave, and the procedures of ECT followed a standardized clinical protocol. Briefly, patients were sedated with intravenous propofol (2–4 mg/kg), and succinylcholine chloride (1–1.5 mg/kg) was used as a muscle relaxant. The participants were provided 100% oxygenation during the ECT procedure, and the electrodes were placed bilaterally over the temporal cortex after the adjustment of duration, power, pulse width, and charge of electric parameters. ECT was performed thrice weekly, and the duration of seizure was maintained at 25 s.

### Hamilton depression rating scale

Hamilton depression rating scale (HAMD) is a 17-item test consisting of eight items scored on a scale from 0 to 2, and nine items from 0 to 4. Higher scores of the 17-item HAMD (HAMD-17) are equal to more severe depression, and HAMD-17 contains five factors, including anxiety/somatization (HAMD-A/S), retardation (HAMD-R), cognitive disorder (HAMD-CD), sleep disorder (HAMD-SLD), and weight (HAMD-W).

### Hamilton anxiety rating scale

Hamilton anxiety rating scale (HAMA) is one of the most common measurements of anxiety in depressed patients, and HAMA is a 14-item test with a five-point Likert scale (0–4) that was provided for distinguishing the severity of anxiety symptoms, where a higher score indicates more severity of a patient’s anxiety. The HAMA-14 consists of somatic anxiety (HAMA-S) and psychological anxiety (HAMA-P) factors.

### Montreal cognitive assessment

The Montreal cognitive assessment (MoCA) is a screening tool for mild cognitive impairment, and its thirty items assess multiple cognitive performances, consisting of visuospatial/executive, naming, attention, language, abstraction, delayed recall, and orientation. The total score of the MoCA is 30, where scores equal to or higher than 26 indicate normal cognitive function.

### Statistical analyses

All statistical analyses were conducted with the SigmaPlot 13.0 and IBM SPSS Statistics 26 software. *Shapiro–Wilk* and *Brown–Forsythe* tests were used to ensure that the data were normality and equal variance, respectively. One-way ANOVA or two-way ANOVA was used to examine the difference among groups, and *Student–Newman–Keuls* method was performed to analyze the pairwise multiple comparison to identify significance between different groups. The data that did not pass the normality and equal variance tests used *Kruskal–Wallis* one-way analysis of variance on ranks for the difference among groups. *Holm–Sidak* or *Dunn*’s method was used for *post hoc* analysis to identify significantly different groups. All statistical analyses were two-tailed, and *p* < 0.05 was considered to be significant. The specific statistical analysis method for each experiment is described in legends. Data are reported as means ± SEM.

## Results

### Demographic and characteristics of the participants

This study included 116 participants aged 53.621 ± 1.202 years, and 31.897% of them were male (*n* = 37). The depression of this hospitalization started with the score of HAMD-17 at 37.155 ± 0.422, and the average of disease course was 29.906 ± 1.561 months. All participants in this study had a follow-up assessments at baseline and weeks 4 and were considered as the treatment cohort. Of those, 22.41% (*n* = 26) were administrated only medication and were classified as control group. The remainder of the cohort, equal to 77.59% (*n* = 90) of the participants, received NIBS treatment, as well as medication. These participants were classed as HF-rTMS, ECT, and HF-rTMS + ECT groups based on the type of NIBS techniques. A schematic of the participant flow is presented in Figure 1. Baseline characteristics of the study cohort are reported in Table 1. No significant differences were identified in sex, age, marital status, education, family history, disease duration, pharmaceutical dosage, as well as in the baseline HAMD, HAMA, and MoCA scores between the four groups (*p* > 0.05, see Table 1).

**TABLE 1:** Demographic and baseline characteristics of groups.

Variable	Control (*n* = 26)	HF-rTMS (*n* = 45)	ECT (*n* = 22)	HF-rTMS + ECT (*n* = 23)	F/H	*p*
Gender (male/female)	10/16	12/33	8/14	7/16	1.307	0.727^a^
Age (years)	54.231 ± 2.599	51.533 ± 1.978	56.727 ± 2.601	54.043 ± 2.665	2.633	0.452
Marital status (single/married/widowed)	1/25/0	2/42/1	0/22/0	0/23/0	3.606	0.863^a^
Education (years)	8.769 ± 0.623	8.311 ± 0.587	8.091 ± 0.993	8.565 ± 0.547	0.672	0.880
Family history (yes/no)	4/22	4/41	7/15	2/21	7.042	0.070^a^
Duration of illness (months)	24.192 ± 2.879	33.156 ± 2.659	26.409 ± 3.205	32.696 ± 3.623	6.425	0.093
First-episode (yes/no)	12/14	16/29	7/15	9/14	1.218	0.749^a^
Antipsychotic dosage	0.327 ± 0.0564	0.425 ± 0.0562	0.498 ± 0.0735	0.386 ± 0.0758	2.761	0.430
Antidepressants dosage	1.302 ± 0.121	1.441 ± 0.114	1.466 ± 0.158	1.682 ± 0.147	3.860	0.277
Kinds of antidepressants (SSRIs/SNRIs/others)	7/15/4	18/17/10	6/10/6	6/13/4	4.386	0.625^a^
Sleeping dosage	8.319 ± 7.668	0.889 ± 0.134	0.782 ± 0.217	0.560 ± 0.166	2.786	0.426
Total score of HAMD-17	36.577 ± 0.783	37.133 ± 0.642	36.727 ± 0.744	38.261 ± 1.312	0.529	0.913
HAMD-A/S	12.423 ± 0.569	13.267 ± 0.382	12.227 ± 0.648	13.478 ± 0.677	3.334	0.343
HAMD-W	1.231 ± 0.101	1.267 ± 0.107	1.045 ± 0.167	1.304 ± 0.159	1.875	0.599
HAMD-CD	8.385 ± 0.356	8.244 ± 0.302	8.409 ± 0.419	8.696 ± 0.489	1.264	0.738
HAMD-R	9.846 ± 0.358	9.911 ± 0.244	10.181 ± 0.404	10.565 ± 0.457	1.773	0.621
HAMD-SD	4.692 ± 0.220	4.422 ± 0.210	4.864 ± 0.40	4.217 ± 0.295	2.854	0.415
Total score of HAMA-14	22.077 ± 0.936	22.467 ± 0.599	23.045 ± 0.725	21.435 ± 0.641	2.092	0.554
HAMA-S	10.154 ± 0.748	10.889 ± 0.422	11.318 ± 0.467	10.609 ± 0.439	1.641	0.650
HAMA-P	11.923 ± 0.440	11.578 ± 0.287	11.727 ± 0.390	10.826 ± 0.337	5.432	0.143
Score of MoCA	26.154 ± 0.297	25.356 ± 0.227	25.955 ± 0.283	25.913 ± 0.235	5.402	0.145
Visuospatial/Executive	4.308 ± 0.144	4.378 ± 0.107	4.500 ± 0.127	4.609 ± 0.104	2.256	0.521
Naming	2.885 ± 0.0639	2.867 ± 0.876	2.818 ± 0.0842	2.739 ± 0.113	1.072	0.784
Attention	5.731 ± 0.245	5.244 ± 0.223	5.591 ± 0.243	5.565 ± 0.234	2.791	0.425
Language	2.885 ± 0.0846	2.778 ± 0.0703	2.727 ± 0.0972	2.609 ± 0.104	4.279	0.233
Abstraction	1.808 ± 0.0788	1.933 ± 0.0376	1.864 ± 0.0749	1.913 ± 0.0601	2.866	0.413
Delayed recall	3.654 ± 0.110	3.489 ± 0.104	3.500 ± 0.158	3.435 ± 0.138	1.553	0.670
Orientation	4.885 ± 0.178	4.667 ± 0.131	4.955 ± 0.192	5.043 ± 0.194	3.325	0.344

Values are presented as mean ± SEM.

^a^Chi-square analysis.

HF-rTMS, high-frequency repetitive transcranial magnetic stimulation; ECT, electroconvulsive therapy; SSRIs, selective serotonin reuptake inhibitors; SNRIs, serotonin and noradrenaline reuptake inhibitors; HAMD-17, 17-item Hamilton depression rating scale; HAMD-A/S, HAMD-anxiety/somatization; HAMD-W, HAMD-weight; HAMD-CD, HAMD-cognitive disorder; HAMD-R, HAMD-retardation; HAMD-SLD, HAMD-sleep disorder; HAMA-14, 14-item Hamilton anxiety rating scale; HAMA-S, HAMA-somatic; HAMA-P, HAMA-psychological; MoCA, Montreal cognitive assessment.

### Improvement of non-invasive brain stimulations on depressive symptoms

**FIGURE 2:**
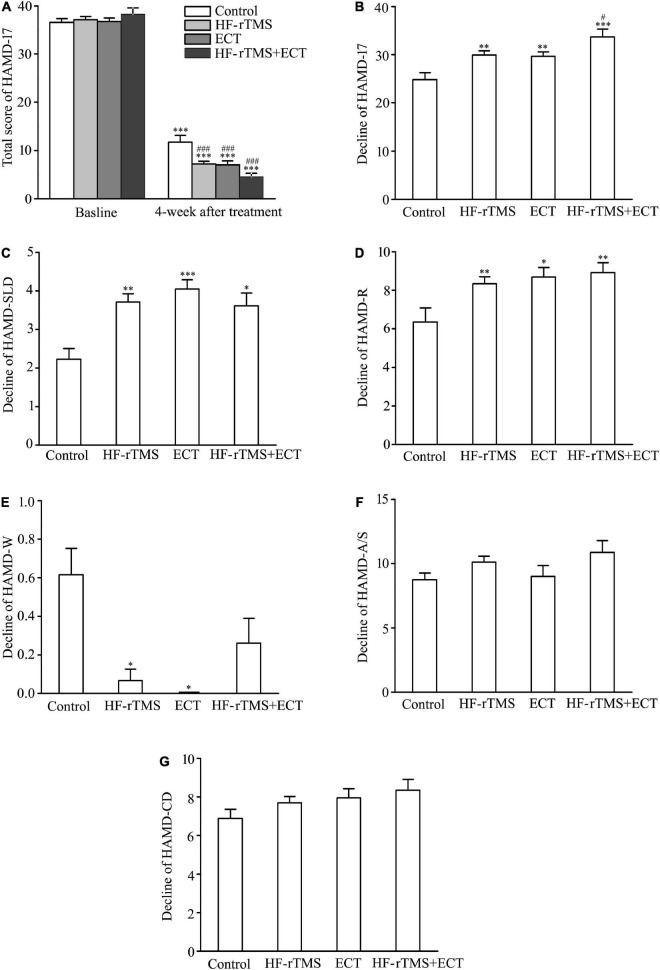
Effect of non-invasive brain stimulation (NIBS) on depression scores measured with HAMD-17 at baseline and 4-week after treatment. **(A)** Total scores of HAMD-17 at baseline and 4 weeks after treatment. ^***^p < 0.001, compared with baseline levels; ^###^*p* < 0.001, compared with control at 4-week after treatment. **(B)** Change in total scores of HAMD-17 from baseline to post-intervention at 4 weeks. ^**^p < 0.01, ^***^p < 0.001, compared with control; ^#^*p* < 0.05, compared with HF-rTMS group. **(C–G)** Change in scores of five factors in HAMD-17; HAMD-SLD **(C)**, HAMD-R **(D)**, HAMD-W **(E)**, HAMD-A/S **(F)**, and HAMD-CD **(G)**. *p < 0.05, ^**^p < 0.01, ***p < 0.001, compared with control **(C–G)**. Graphs show mean ± SEM.

Hamilton depression rating scale-17 (HAMD-17) total scores, indicating subjective depressive symptoms, significantly decreased in control, HF-rTMS, ECT, and HF-rTMS + ECT groups as treatment progressed, F = 2158.437, DF = 1, *p* < 0.001, and the HAMD-17 total scores at 4 weeks after intervention were deferent between the four groups, F = 3.468, DF = 3, *p* = 0.017. The group × time interaction in the ANOVA comparing control, HF-rTMS, ECT, and HF-rTMS + ECT groups was significant, F = 7.611, DF = 3, *p* < 0.001. Multiple comparisons analyses showed a statistical significance between baseline and 4-week after treatment in the control, HF-rTMS, ECT, and HF-rTMS + ECT groups (see Figure 2A), and there was a significance at the end of 4-week after treatment between control group and the other groups (control *vs*. HF-rTMS, *q* = 5.644, *p* < 0.001; control *vs*. ECT, *q* = 4.962, *p* = 0.001; control *vs*. HF-rTMS + ECT, *q* = 7.658, *p* < 0.001; see Figure 2A). However, there was no significance among the HF-rTMS, ECT, and HF-rTMS + ECT groups though HF-rTMS + ECT group had lower total score of HAMD-17 than HF-rTMS or ECT group (HF-rTMS *vs*. HF-rTMS + ECT group, *q* = 3.128, *p* = 0.069; ECT *vs*. HF-rTMS + ECT group, *q* = 2.531, *p* = 0.074; see Figure 2A). To confirm the effect of HF-rTMS, ECT, and HF-rTMS + ECT treatment on depression, we analyzed the decline in the HAMD-17 total scores from baseline to post-intervention at 4 weeks. The results showed that there was a statistically significant difference among the four groups (F = 8.151, DF = 3, *p* < 0.001; see Figure 2B), and multiple comparison analyses showed that HF-rTMS, ECT, or HF-rTMS + ECT treatment accelerated the reduction of depression (control *vs*. HF-rTMS, *q* = 4.633, *p* = 0.004; control *vs*. ECT, *q* = 3.746, *p* = 0.009; control *vs*. HF-rTMS + ECT, *q* = 6.913, *p* < 0.001). In particular, HF-rTMS combined ECT method promoted depression better than HF-rTMS treatment did (HF-rTMS *vs*. HF-rTMS + ECT group, *q* = 3.268, *p* = 0.023; ECT *vs*. HF-rTMS + ECT group, *q* = 2.977, *p* = 0.091; see Figure 2B).

Hamilton depression rating scale-17 includes five factors: sleep disorder (HAMD-SLD), retardation (HAMD-R), weight (HAMD-W), anxiety/somatization (HAMD-A/S), and cognitive disorder (HAMD-CD). Of the five factors, one-way ANOVA analysis showed that there was a statistical difference in the decline scores of HAMD-SLD, HAMD-R, and HAMD-W factors from baseline to post-intervention at 4 weeks among the control, HF-rTMS, ECT, and HF-rTMS + ECT groups (HAMD-SLD, H = 15.592, *p* < 0.001; HAMD-R, F = 4.629, DF = 3, *p* = 0.004; HAMD-W, H = 26.238, *p* < 0.001; see Figures 2C–E), while the other two factors did not reach statistical significance (see Figures 2F,G). In addition, multiple comparison analyses showed that HF-rTMS, ECT, or HF-rTMS + ECT ameliorated HAMD-SLD and HAMD-R better than the control group, while they resisted the effect of medication on HAMD-W (HF-rTMS *vs.* control, Q = 3.725, *p* = 0.001; ECT *vs.* control, Q = 3.805, *p* < 0.001; HF-rTMS + ECT *vs.* control, Q = 3.011, *p* = 0.016 for HAMD-SLD, see Figure 2C; HF-rTMS *vs.* control, q = 4.150, *p* = 0.004; ECT *vs.* control, q = 4.148, *p* = 0.011; HF-rTMS + ECT *vs.* control, q = 4.613, *p* = 0.008 for HAMD-R, see Figure 2D; HF-rTMS *vs.* control, Q = 3.092, *p* = 0.012; ECT *vs.* control, Q = 2.916, *p* = 0.012; HF-rTMS + ECT *vs.* control, Q = 1.876, *p* = 0.364 for HAMD-W, see Figure 2E). Taken together, non-invasive brain stimulations (HF-rTMS, ECT, or HF-rTMS + ECT) have the efficacy on sleep disturbances, retardation, and weight factors.

### Effect of non-invasive brain stimulations on anxiety

**FIGURE 3:**
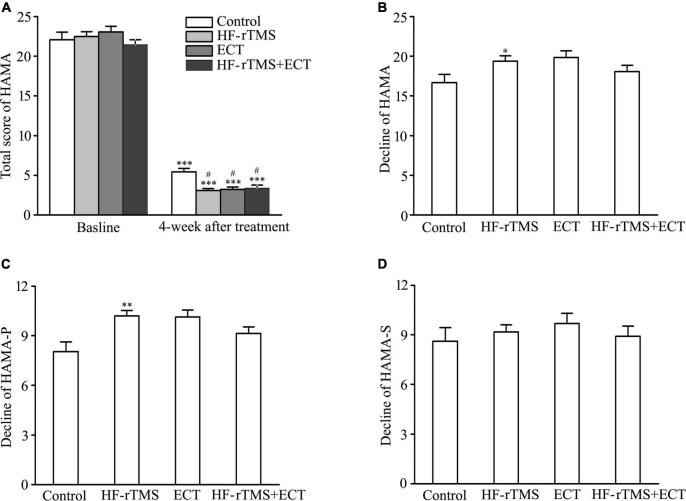
Effect of non-invasive brain stimulation (NIBS) on anxiety detected by HAMA-14 at baseline and 4-week after treatment. **(A)** Total scores of HAMA-14 at baseline and 4 weeks after treatment. ^***^p < 0.001, compared with baseline levels; ^#^*p* < 0.001, compared with control at 4-week after treatment. **(B)** Decline in total scores of HAMA-14 from baseline to post-intervention at 4 weeks. *p < 0.05, compared with control. **(C,D)** Decline in scores of two factors in HAMA-14. HAMA-P **(C)**, HAMA-S **(D)**. ^**^p < 0.01, compared with control. Graphs show mean ± SEM.

Hamilton anxiety rating scale-14 total scores, indicating anxiety severity, significantly decreased in control, HF-rTMS, ECT, and HF-rTMS + ECT groups as treatment progressed, F = 1949.184, DF = 1, *p* < 0.001. The group × time interaction in the ANOVA comparing control, HF-rTMS, ECT, and HF-rTMS + ECT groups was significant, F = 2.945, DF = 3, *p* = 0.034. Multiple comparisons analyses showed a statistical significance between baseline and 4-week after treatment in the control, HF-rTMS, ECT, and HF-rTMS + ECT groups (baseline *vs*. 4 weeks after treatment, q = 27.754, *p* < 0.001 in control; q = 42.485, *p* < 0.001 in HF-rTMS; q = 30.381, *p* < 0.001 in ECT; q = 28.282, *p* < 0.001 in HF-rTMS + ECT; see Figure 3A), and there was a significance at the end of 4-week after treatment between control group and the other groups (control *vs*. HF-rTMS, *q* = 4.380, *p* = 0.011; control *vs*. ECT, *q* = 3.504, *p* = 0.035; control *vs*. HF-rTMS + ECT, *q* = 3.281, *p* = 0.020; see Figure 3A). To confirm the effect of HF-rTMS, ECT, and HF-rTMS + ECT treatment on anxiety, we analyzed the decline in the HAMA-14 total scores from baseline to post-intervention at 4 weeks. The results of one-way ANOVA showed that there was a statistically significant difference among the four groups (F = 2.744, DF = 3, *p* = 0.046; see Figure 3B), and multiple comparison analyses showed that HF-rTMS treatment accelerated the reduction of anxiety (control *vs*. HF-rTMS, *q* = 3.488, *p* = 0.040, see Figure 3B). However, there was no significance among the HF-rTMS, ECT, and HF-rTMS + ECT groups (see Figures 3A,B).

Hamilton anxiety rating scale-14 total items divide into the psychological (HAMA-P) and somatic (HAMA-S) factors. One-way ANOVA analysis showed that there was a statistical difference in the decline score of HAMA-P from baseline to post-intervention at 4 weeks among the control, HF-rTMS, ECT, and HF-rTMS + ECT groups (H = 12.853, *p* = 0.005; see Figure 3C), while the decline score of HAMA-S did not reach statistical significance (see Figure 3D). In addition, multiple comparison analyses showed that HF-rTMS ameliorated HAMA-P (HF-rTMS *vs.* control, Q = 3.202, *p* = 0.008, see Figure 3C). Therefore, HF-rTMS alleviated psychological anxiety in major depression.

### Improvement of high-frequency repetitive transcranial magnetic stimulation on the cognitive impairment induced by electroconvulsive therapy

**FIGURE 4:**
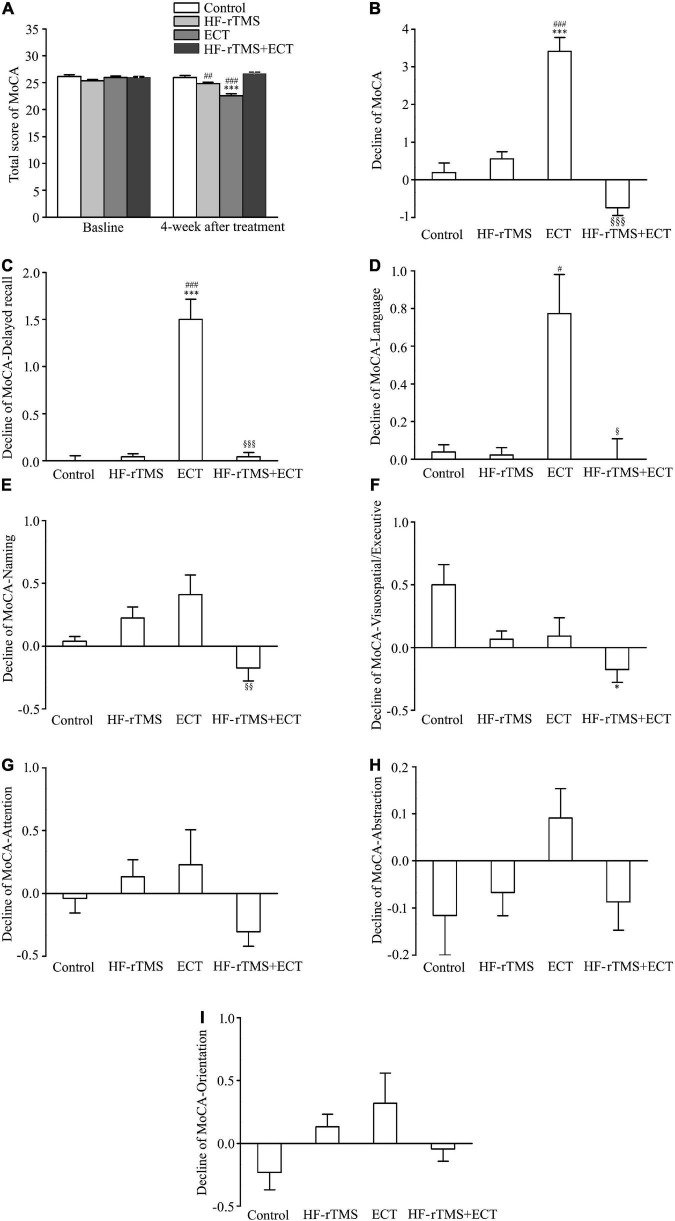
High-frequency repetitive transcranial magnetic stimulation (HF-rTMS) reversed the cognitive impairment evoked by ECT. **(A)** Total scores of MoCA at baseline and 4 weeks after treatment. ^***^p < 0.001, compared with baseline levels; ^##^*p* < 0.01, ^###^*p* < 0.001, compared with control at 4-week after treatment. **(B)** Decline in total scores of MoCA from baseline to post-intervention at 4 weeks. ^***^p < 0.001, compared with control; ^###^*p* < 0.001, compared with HF-rTMS. ^§§§^ *p* < 0.001, compared with ECT. **(C–I)** Decline in scores of five performances in MoCA; delayed recall **(C)**, language **(D)**, naming **(E)**, visuospatial/executive **(F)**, attention **(G)**, abstraction **(H)**, and orientation **(I)**. *p < 0.05, ^***^p < 0.001, compared with control; ^#^*p* < 0.05, ^##^*p* < 0.01, ^###^*p* < 0.001, compared with HF-rTMS; ^§^*p* < 0.05, ^§§^*p* < 0.01, ^§§§^*p* < 0.001, compared with ECT **(C–F)**. Graphs show mean ± SEM.

The MoCA, which is known to be highly sensitive in identifying patients with mild cognitive impairment from the normal population (33), was used to test the cognitive side effects of ECT. MoCA scores were significantly different among control, HF-rTMS, ECT, and HF-rTMS + ECT groups as treatment progressed, F = 14.120, DF = 1, *p* < 0.001, and the scores at 4 weeks after intervention were deferent between the four groups, F = 15.162, DF = 3, *p* < 0.001. The group × time interaction in the ANOVA comparing control, HF-rTMS, ECT, and HF-rTMS + ECT groups was significant, F = 13.041, DF = 3, *p* < 0.001. Multiple comparisons analyses showed a statistical significance between baseline and 4-week after treatment only in the ECT group (baseline *vs*. 4 weeks after treatment, q = 9.617, *p* < 0.001 in ECT, see Figure 4A), and there was a significance at the end of 4-week after treatment among the four groups except between control and HF-rTMS + ECT (control *vs*. HF-rTMS, *q* = 4.010, *p* = 0.005; control *vs*. ECT, *q* = 10.030, *p* < 0.001; control *vs*. HF-rTMS + ECT, *q* = 2.052, *p* = 0.147; HF-rTMS *vs*. ECT, *q* = 7.371, *p* < 0.001; ECT *vs*. HF-rTMS + ECT, *q* = 11.712, *p* < 0.001; see Figure 4A). To confirm the side effect of non-invasive brain stimulations on cognition, we analyzed the decline in MoCA scores from baseline to post-intervention at 4 weeks. The results of one-way ANOVA showed that there was a statistically significant difference among the four groups (H = 52.535, *p* < 0.001; see Figure 4B), and multiple comparison analyses showed that ECT evoked cognitive impairment, which was reversed by HF-rTMS (control *vs*. ECT, *Q* = 5.036, *p* < 0.001; control *vs*. HF-rTMS + ECT, *Q* = 2.116, *p* = 0.206; HF-rTMS *vs*. ECT, *Q* = 4.564, *p* < 0.001; ECT *vs*. HF-rTMS + ECT, *Q* = 6.922, *p* < 0.001; see Figure 4B).

The MoCA assesses multiple cognitive performances, consisting of visuospatial/executive function, naming, attention, language, abstraction, delayed recall, and orientation. Of these functions, one-way ANOVA analysis showed that there was a statistical difference in the decline scores of delayed recall, language, naming, visuospatial/executive, and attention from baseline to post-intervention at 4 weeks among the control, HF-rTMS, ECT, and HF-rTMS + ECT (delayed recall, H = 65.363, *p* < 0.001; language, H = 24.964, *p* < 0.001; naming, H = 16.082, *p* = 0.001; visuospatial/executive, H = 12.818, *p* = 0.005; attention, H = 8.310, *p* = 0.040; see Figures 4C–G), while the other two functions did not reach statistical significance (see Figures 4H,I). Multiple comparison analyses showed that ECT evoked the delayed recall impairment, which was reversed by HF-rTMS (control *vs*. ECT, *Q* = 4.668, *p* < 0.001; control *vs*. HF-rTMS + ECT, *Q* = 0.216, *p* = 1.000; HF-rTMS *vs*. ECT, *Q* = 4.955, *p* < 0.001; ECT *vs*. HF-rTMS + ECT group, *Q* = 4.327, *p* < 0.001; see Figure 4C). For language, there was significant difference among HF-rTMS, ECT, and HF-rTMS + ECT groups (HF-rTMS *vs*. ECT, *Q* = 2.846, *p* = 0.027; ECT *vs*. HF-rTMS + ECT, *Q* = 2.781, *p* = 0.032; see Figure 4D). Difference between ECT and HF-rTMS + ECT was found in naming function (Q = 2.870, *p* = 0.025; see Figure 4E), and difference between control and HF-rTMS + ECT was found in visuospatial/executive function (Q = 2.805, *p* = 0.030; see Figure 4F). However, there was no difference among the four groups in attention, abstraction, and orientation factors (see Figures 4F–I).

## Discussion

The main aim of the retrospective study was to evaluate the clinical efficacy of NIBS on anti-depressant treatment and its impact on cognitive function. For the anti-depressant efficacy, there was no significant difference in HAMD scores among HF-rTMS, ECT, and HF-rTMS + ECT groups at baseline and post-treatment levels, but there was a significant decrease in HAMD scores compared with baseline levels and control group, confirming that NIBS has the anti-depressant efficacy. In terms of cognitive dimension, significance in MoCA scores between baseline and post-treatment levels was only found in ECT group, and HF-rTMS reversed the cognitive impairment induced by ECT.

Electroconvulsive therapy, involving a generalized controlled seizure, produced by a series of short electric current bursts delivered through electrodes to the brain, is the oldest NIBS method utilized in the treatment of severe depression (20, 24). In the retrospective study, the cohort of 116 patients with HAMD scores at 26 or greater, identified as severe depression, between the ages of 18 and 75, and the course of MDD between 12 and 64 months were enrolled in the intervention and provided 4-week follow-up assessment in the program (Figure 1). In contrast to ECT, rTMS, utilizing non-convulsive focal stimulation of the brain through a time-varying electromagnetic field, is one of the newer NIBS methods (27). Clinically, studies proved the anti-depressant efficacy of HF-rTMS over DLPFC, which has been approved by the Food and Drug Administration in USA and later in the EU for the treatment of depression (34–37). However, it is controversial whether ECT is more effective than HF-rTMS in the treatment of MDD. Some studies stated that ECT is more effective than rTMS in the treatment of MDD (38, 39), but meta-analysis, based on 25 studies consisting of 1,288 individuals with MDD, showed that ECT was non-significantly more efficacious than HF-rTMS in anti-depressant effect (30).

Hamilton depression scale is the most commonly used in clinical evaluation of depression (40). In the study, the total score of HAMD-17 was measured at baseline and at the end of 4-week treatment. At baseline, there was no difference among control, HF-rTMS, ECT, and HF-rTMS + ECT groups (Table 1), and the total score of HAMD-17 at 4-week after treatment decreased significantly in the four groups compared with the baseline because medication was kept constant as well in all participants (Figure 2A). Besides, NIBS treatment ameliorated depressive symptom significantly though there was no statistically difference between HF-rTMS and ECT (Figures 2A,B). To confirm the result, we compared the decline in HAMD-17 scores from baseline to post-treatment among control, HF-rTMS, ECT, and HF-rTMS + ECT groups. The data showed that there was no difference between HF-rTMS and ECT (Figures 2A,B). Therefore, our results verified that ECT was non-significantly more efficacious than HF-rTMS in anti-depressant effect (30). Furthermore, our study also tested whether ECT can improve the anti-depressant effect of HF-rTMS, because ECT and TMS are different methods of NIBS. Interestingly, our results showed that ECT promoted the anti-depressant efficacy of HF-rTMS significantly (Figure 2B). HAMD-17 includes HAMD-A/S, HAMD-W, HAMD-CD, HAMD-R, and HAMD-SLD, and we assessed the scores of its five factors at pre- and post-treatment. The results showed that NIBS only affected HAMD-SLD, HAMD-R, and HAMD-W though there was no difference between HF-rTMS and ECT (Figures 2C–G). Taken together, the retrospective study confirmed that HF-rTMS has the same anti-depressant efficacy as ECT *via* HAMD-SLD, HAMD-R, and HAMD-W. Furthermore, the combination of HF-rTMS with ECT was better than HF-rTMS or ECT alone in the treatment of depressive symptoms, though low-frequency rTMS inhibits the anti-depressive effect of ECT (27). These findings imply that HF-rTMS has the same anti-depressant efficacy as ECT, but combination of HF-rTMS and ECT is preferred for the treatment of patients with severe depression.

Anxiety and depression have been considered as two distinct entities according to the diagnostic criteria (41), but anxiety symptoms are common in depressed individuals across lifespan (42–45). Thus, scores of HAMA-14 and its two factors were assessed to examine the effect of NIBS in MDD. At baseline, there was no difference among control, HF-rTMS, ECT, and HF-rTMS + ECT groups in total scores of HAMA-14, and scores of HAMA-P and HAMA-S (Table 1). However, the total scores of HAMA-14 at 4-week after treatment decreased significantly in the four groups compared with the baseline because medication was kept constant as well in all participants (Figure 3A). Besides, NIBS treatment improved anxious symptom significantly though there was no statistically difference between HF-rTMS and ECT (Figure 3A). To confirm the result, we compared the decline in HAMA-14 scores and scores of its factors from baseline to post-treatment among control, HF-rTMS, ECT, and HF-rTMS + ECT groups. The results showed that HF-rTMS improved HAMA-P though there was no statistically difference among control, HF-rTMS, ECT, and HF-rTMS + ECT groups in total scores of HAMA-14 (Figures 3B–D). Therefore, HF-rTMS should be selected for MDD patients with anxiety.

Montreal cognitive assessment is known to distinguish patients with mild cognitive impairment from the normal population, and it exhibits higher sensitivity in detecting cognitive decline than other common clinical screening tool, such as the Mini-Mental State Examination (33, 46, 47). Therefore, we examined the cognitive side effects of NIBS with MoCA in the present study. Our data showed that ECT, not HF-rTMS, impairs cognitive function with the decline of MoCA scores (Figures 4A,B). The results are consistent with previous studies, which have found that ECT potentially has detrimental cognitive side effects, including amnesia and slowing of reaction times (48, 49). Furthermore, HF-rTMS could reverse the cognitive impairment induced by ECT (Figures 4A,B). The MoCA assesses multiple cognitive performances, consisting of visuospatial/executive function, naming, attention, language, abstraction, delayed recall, and orientation. We also tested the decline of these cognitive performances and found that ECT, not HF-rTMS, impacted on delayed recall and language specifically (Figures 4C–I). In particular, HF-rTMS could reverse the decline of the two cognitive performances evoked by ECT. These data further suggest that HF-rTMS or combination of HF-rTMS and ECT is preferred for the treatment of MDD patients.

In sum, the retrospective study has revealed that HF-rTMS may be an efficacious treatment for depression with an effect size similar to ECT, and it benefits anxiety and cognition impairment evoked by ECT. Thus, HF-rTMS and combination of HF-rTMS and ECT were the treatment option for severe depression.

## Limitations

The design of the current study was retrospective, and the sample size is small. A larger and more representative sample is needed to confirm our findings and distinguish the efficacy of HF-rTMS and ECT in female and male MDD patients because fMRI findings have revealed that male MDD patients exhibited increased neural stress responses in the DLPFC and frontoparietal network (PFN), while female MDD patients explored less deactivation in limbic-striatal regions including the amygdala, hippocampus, and nucleus accumbens (NAc) (50). Moreover, there was a small difference in the variables of family history and duration of illness (Table 1). In addition to that, the study only examined the 4-week follow-up data, and future research is certainly needed to explore the long-term efficacy of HF-rTMS and ECT in MDD patients.

## Data availability statement

The raw data supporting the conclusions of this article will be made available by the authors, without undue reservation.

## Ethics statement

The studies involving human participants were reviewed and approved by Ethics Committee of Nantong Fourth People’s Hospital in China (Approval Number: 2020K007). The patients/participants provided their written informed consent to participate in this study.

## Author contributions

XC and HS contributed to conception and design of the study and performed the statistical analysis. TZ, XS, QY, PZ, HZ, FJ, CL, and YL collected the data. WL and JX organized the database. HS wrote the manuscript. All authors contributed to the article and approved the submitted version.
